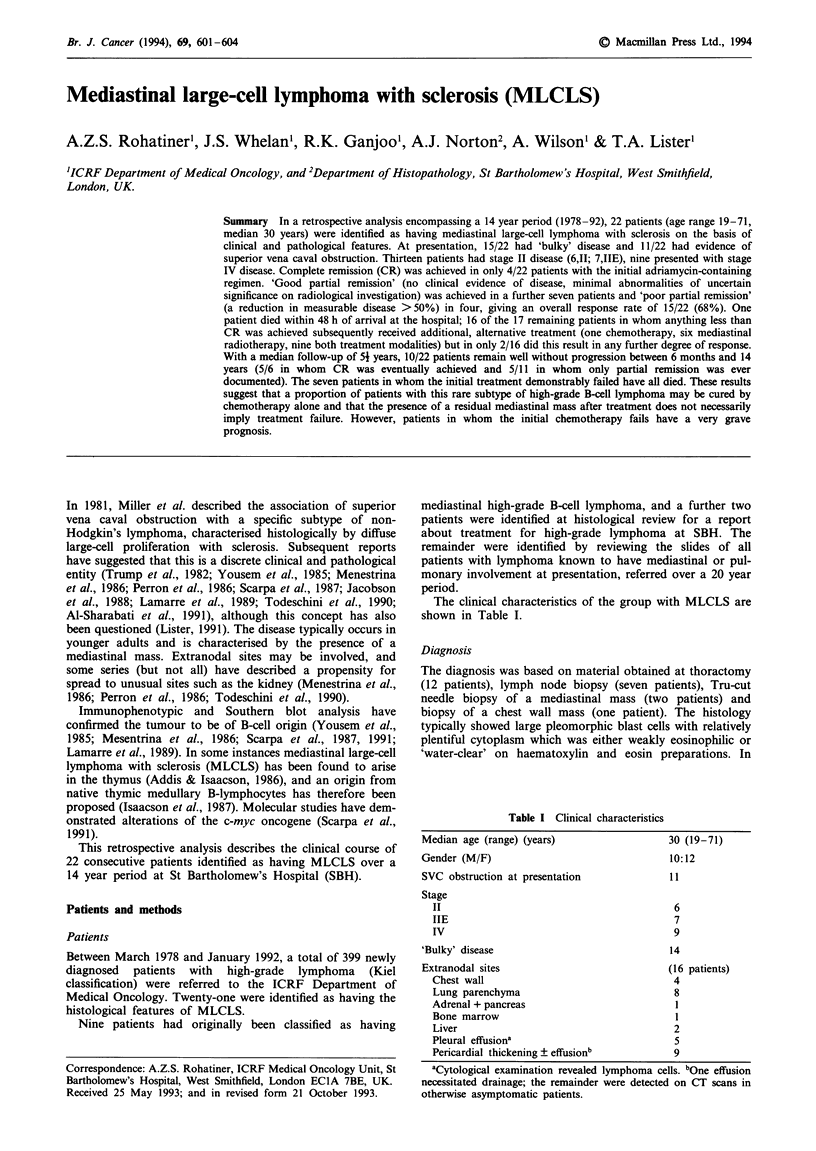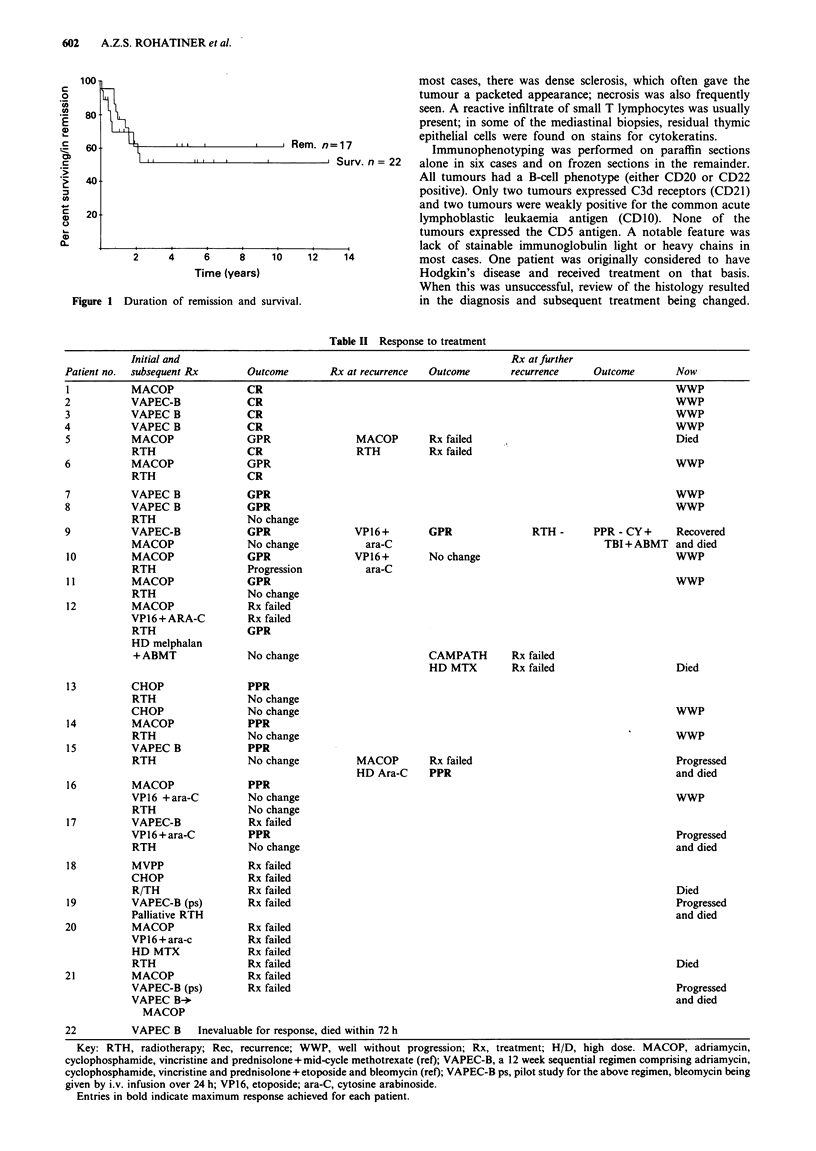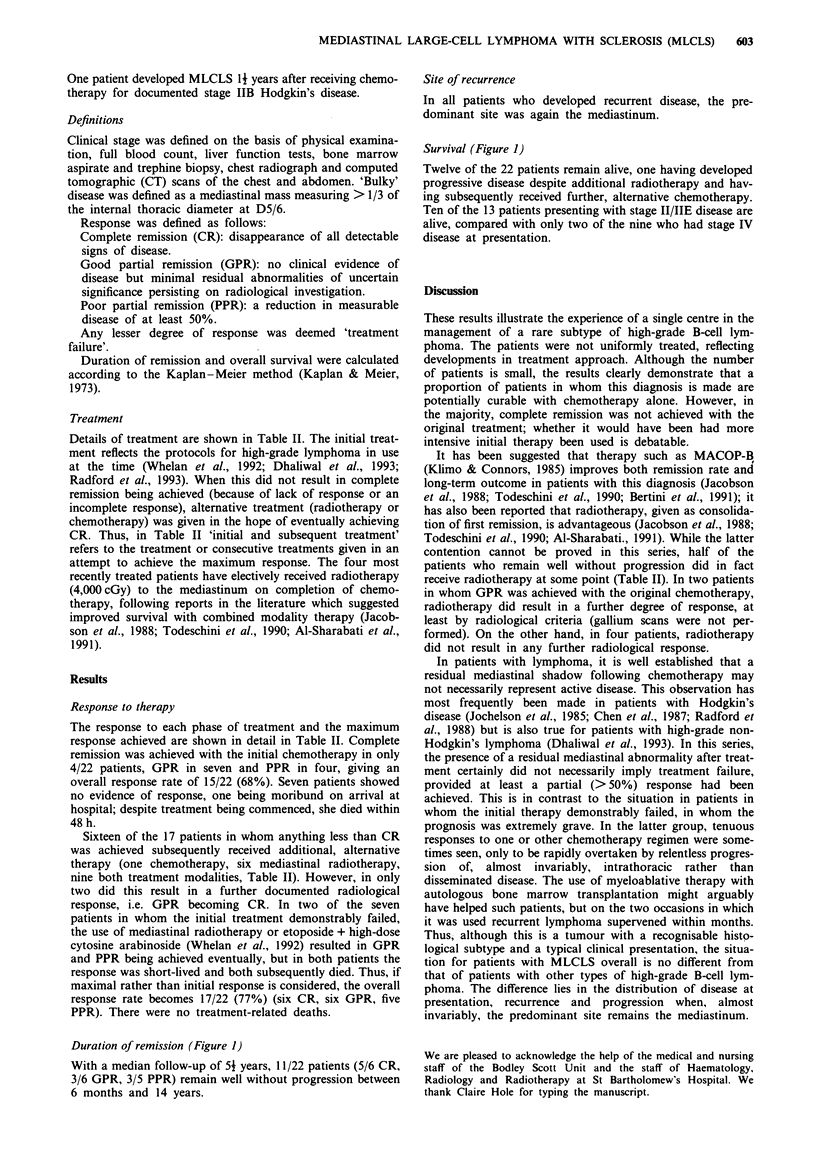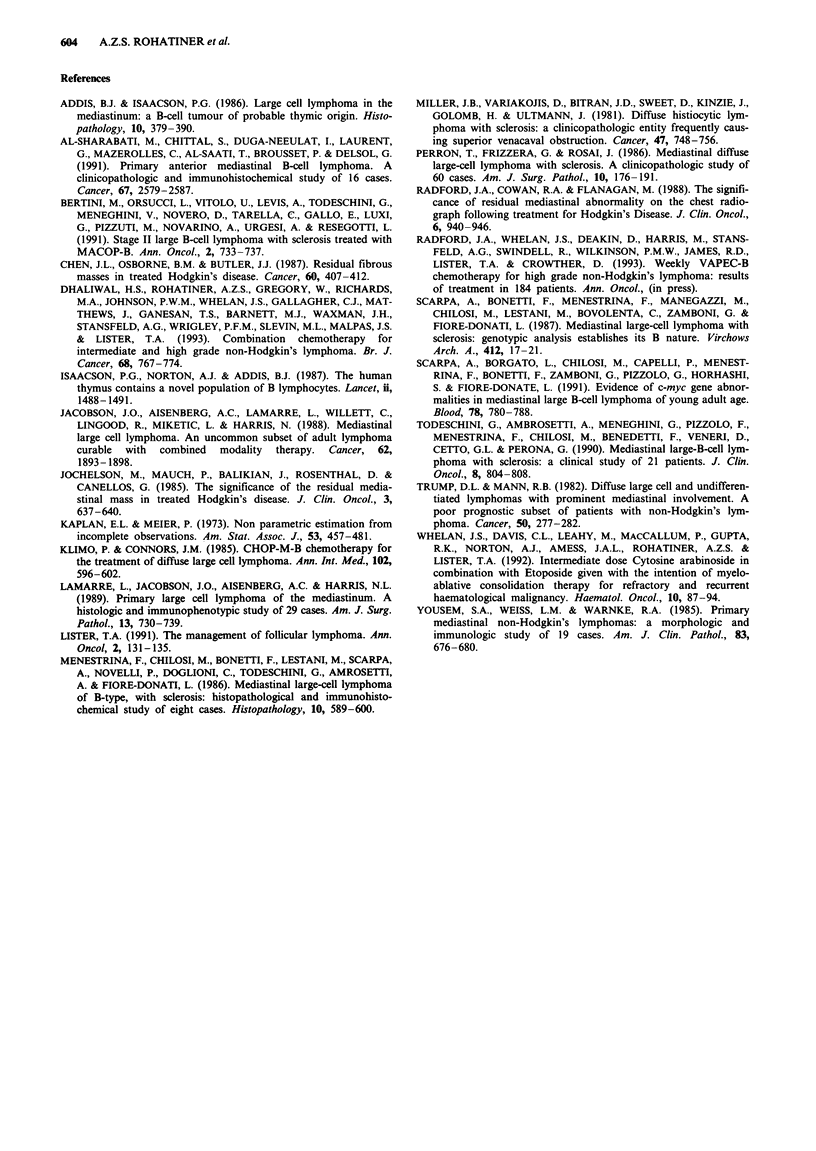# Mediastinal large-cell lymphoma with sclerosis (MLCLS).

**DOI:** 10.1038/bjc.1994.111

**Published:** 1994-03

**Authors:** A. Z. Rohatiner, J. S. Whelan, R. K. Ganjoo, A. J. Norton, A. Wilson, T. A. Lister

**Affiliations:** ICRF Department of Medical Oncology, St Bartholomew's Hospital, West Smithfield, London, UK.

## Abstract

In a retrospective analysis encompassing a 14 year period (1978-92), 22 patients (age range 19-71, median 30 years) were identified as having mediastinal large-cell lymphoma with sclerosis on the basis of clinical and pathological features. At presentation, 15/22 had 'bulky' disease and 11/22 had evidence of superior vena caval obstruction. Thirteen patients had stage II disease (6,II; 7,IIE), nine presented with stage IV disease. Complete remission (CR) was achieved in only 4/22 patients with the initial adriamycin-containing regimen. 'Good partial remission' (no clinical evidence of disease, minimal abnormalities of uncertain significance on radiological investigation) was achieved in a further seven patients and 'poor partial remission' (a reduction in measurable disease > 50%) in four, giving an overall response rate of 15/22 (68%). One patient died within 48 h of arrival at the hospital; 16 of the 17 remaining patients in whom anything less than CR was achieved subsequently received additional, alternative treatment (one chemotherapy, six mediastinal radiotherapy, nine both treatment modalities) but in only 2/16 did this result in any further degree of response. With a median follow-up of 5 1/2 years, 10/22 patients remain well without progression between 6 months and 14 years (5/6 in whom CR was eventually achieved and 5/11 in whom only partial remission was ever documented). The seven patients in whom the initial treatment demonstrably failed have all died. These results suggest that a proportion of patients with this rare subtype of high-grade B-cell lymphoma may be cured by chemotherapy alone and that the presence of a residual mediastinal mass after treatment does not necessarily imply treatment failure. However, patients in whom the initial chemotherapy fails have a very grave prognosis.


Br. J. Cancer (1994), 69, 601-604 © Macmillan Press Ltd., 1994

### Mediastinal large-cell lymphoma with sclerosis (MLCLS)

A.Z.S. Rohatiner[1], J.S. Whelan[1], R.K. Ganjoo[1], A.J. Norton[2], A. Wilson[1] & T.A. Lister[1]

[1]*ICRF Department of Medical Oncology, and* [2]*Department of Histopathology, St Bartholomew's Hospital, West Smithfield, London, UK.*

**Summary** In a retrospective analysis encompassing a 14 year period (1978-92), 22 patients (age range 19-71, median 30 years) were identified as having mediastinal large-cell lymphoma with sclerosis on the basis of clinical and pathological features. At presentation, 15/22 had 'bulky' disease and 11/22 had evidence of superior vena caval obstruction. Thirteen patients had stage II disease (6,II; 7,IIE), nine presented with stage IV disease. Complete remission (CR) was achieved in only 4/22 patients with the initial adriamycin-containing regimen. 'Good partial remission' (no clinical evidence of disease, minimal abnormalities of uncertain significance on radiological investigation) was achieved in a further seven patients and 'poor partial remission' (a reduction in measurable disease >50%) in four, giving an overall response rate of 15/22 (68%). One patient died within 48 h of arrival at the hospital; 16 of the 17 remaining patients in whom anything less than CR was achieved subsequently received additional, alternative treatment (one chemotherapy, six mediastinal radiotherapy, nine both treatment modalities) but in only 2/16 did this result in any further degree of response. With a median follow-up of 5¼ years, 10/22 patients remain well without progression between 6 months and 14 years (5/6 in whom CR was eventually achieved and 5/11 in whom only partial remission was ever documented). The seven patients in whom the initial treatment demonstrably failed have all died. These results suggest that a proportion of patients with this rare subtype of high-grade B-cell lymphoma may be cured by chemotherapy alone and that the presence of a residual mediastinal mass after treatment does not necessarily imply treatment failure. However, patients in whom the initial chemotherapy fails have a very grave prognosis.

In 1981, Miller *et al.* described the association of superior vena caval obstruction with a specific subtype of non-Hodgkin's lymphoma, characterised histologically by diffuse large-cell proliferation with sclerosis. Subsequent reports have suggested that this is a discrete clinical and pathological entity (Trump *et al.*, 1982; Yousem *et al.*, 1985; Menestrina *et al.*, 1986; Perron *et al.*, 1986; Scarpa *et al.*, 1987; Jacobson *et al.*, 1988; Lamarre *et al.*, 1989; Todeschini *et al.*, 1990; Al-Sharabati *et al.*, 1991), although this concept has also been questioned (Lister, 1991). The disease typically occurs in younger adults and is characterised by the presence of a mediastinal mass. Extranodal sites may be involved, and some series (but not all) have described a propensity for spread to unusual sites such as the kidney (Menestrina *et al.*, 1986; Perron *et al.*, 1986; Todeschini *et al.*, 1990).

Immunophenotypic and Southern blot analysis have confirmed the tumour to be of B-cell origin (Yousem *et al.*, 1985; Mesentrina *et al.*, 1986; Scarpa *et al.*, 1987, 1991; Lamarre *et al.*, 1989). In some instances mediastinal large-cell lymphoma with sclerosis (MLCLS) has been found to arise in the thymus (Addis & Isaacson, 1986), and an origin from native thymic medullary B-lymphocytes has therefore been proposed (Isaacson *et al.*, 1987). Molecular studies have demonstrated alterations of the c-*myc* oncogene (Scarpa *et al.*, 1991).

This retrospective analysis describes the clinical course of 22 consecutive patients identified as having MLCLS over a 14 year period at St Bartholomew's Hospital (SBH).

### Patients and methods

### *Patients*

Between March 1978 and January 1992, a total of 399 newly diagnosed patients with high-grade lymphoma (Kiel classification) were referred to the ICRF Department of Medical Oncology. Twenty-one were identified as having the histological features of MLCLS.

Nine patients had originally been classified as having mediastinal high-grade B-cell lymphoma, and a further two patients were identified at histological review for a report about treatment for high-grade lymphoma at SBH. The remainder were identified by reviewing the slides of all patients with lymphoma known to have mediastinal or pulmonary involvement at presentation, referred over a 20 year period.

The clinical characteristics of the group with MLCLS are shown in Table I.

### *Diagnosis*

The diagnosis was based on material obtained at thoractomy (12 patients), lymph node biopsy (seven patients), Tru-cut needle biopsy of a mediastinal mass (two patients) and biopsy of a chest wall mass (one patient). The histology typically showed large pleomorphic blast cells with relatively plentiful cytoplasm which was either weakly eosinophilic or 'water-clear' on haematoxylin and eosin preparations. In

**Table I** Clinical characteristics

| | |
|---|---|
| Median age (range) (years) | 30 (19-71) |
| Gender (M/F) | 10:12 |
| SVC obstruction at presentation | 11 |
| Stage | |
| II | 6 |
| IIE | 7 |
| IV | 9 |
| 'Bulky' disease | 14 |
| Extranodal sites | (16 patients) |
| Chest wall | 4 |
| Lung parenchyma | 8 |
| Adrenal + pancreas | 1 |
| Bone marrow | 1 |
| Liver | 2 |
| Pleural effusion[a] | 5 |
| Pericardial thickening ± effusion[b] | 9 |

[a]Cytological examination revealed lymphoma cells. [b]One effusion necessitated drainage; the remainder were detected on CT scans in otherwise asymptomatic patients.

Correspondence: A.Z.S. Rohatiner, ICRF Medical Oncology Unit, St Bartholomew's Hospital, West Smithfield, London EC1A 7BE, UK.
Received 25 May 1993; and in revised form 21 October 1993.

**Figure 1** Duration of remission and survival.

most cases, there was dense sclerosis, which often gave the tumour a packeted appearance; necrosis was also frequently seen. A reactive infiltrate of small T lymphocytes was usually present; in some of the mediastinal biopsies, residual thymic epithelial cells were found on stains for cytokeratins.

Immunophenotyping was performed on paraffin sections alone in six cases and on frozen sections in the remainder. All tumours had a B-cell phenotype (either CD20 or CD22 positive). Only two tumours expressed C3d receptors (CD21) and two tumours were weakly positive for the common acute lymphoblastic leukaemia antigen (CD10). None of the tumours expressed the CD5 antigen. A notable feature was lack of stainable immunoglobulin light or heavy chains in most cases. One patient was originally considered to have Hodgkin's disease and received treatment on that basis. When this was unsuccessful, review of the histology resulted in the diagnosis and subsequent treatment being changed.

**Table II** Response to treatment

| *Patient no.* | *Initial and subsequent Rx* | *Outcome* | *Rx at recurrence* | *Outcome* | *Rx at further recurrence* | *Outcome* | *Now* |
|---|---|---|---|---|---|---|---|
| 1 | MACOP | **CR** | | | | | WWP |
| 2 | VAPEC-B | **CR** | | | | | WWP |
| 3 | VAPEC B | **CR** | | | | | WWP |
| 4 | VAPEC B | **CR** | | | | | WWP |
| 5 | MACOP | GPR | MACOP | Rx failed | | | Died |
| | RTH | **CR** | RTH | Rx failed | | | |
| 6 | MACOP | GPR | | | | | WWP |
| | RTH | **CR** | | | | | |
| 7 | VAPEC B | **GPR** | | | | | WWP |
| 8 | VAPEC B | **GPR** | | | | | WWP |
| | RTH | No change | | | | | |
| 9 | VAPEC-B | **GPR** | VP16+ ara-C | **GPR** | RTH - | PPR - CY+ TBI+ABMT | Recovered and died |
| | MACOP | No change | | | | | |
| 10 | MACOP | **GPR** | VP16+ ara-C | No change | | | WWP |
| | RTH | Progression | | | | | |
| 11 | MACOP | **GPR** | | | | | WWP |
| | RTH | No change | | | | | |
| 12 | MACOP | Rx failed | | | | | |
| | VP16+ARA-C | Rx failed | | | | | |
| | RTH | **GPR** | | | | | |
| | HD melphalan +ABMT | No change | | CAMPATH | Rx failed | | |
| | | | | HD MTX | Rx failed | | Died |
| 13 | CHOP | **PPR** | | | | | |
| | RTH | No change | | | | | |
| | CHOP | No change | | | | | WWP |
| 14 | MACOP | **PPR** | | | | | |
| | RTH | No change | | | | | WWP |
| 15 | VAPEC B | **PPR** | | | | | |
| | RTH | No change | MACOP | Rx failed | | | Progressed |
| | | | HD Ara-C | **PPR** | | | and died |
| 16 | MACOP | **PPR** | | | | | |
| | VP16 +ara-C | No change | | | | | WWP |
| | RTH | No change | | | | | |
| 17 | VAPEC-B | Rx failed | | | | | |
| | VP16+ara-C | **PPR** | | | | | Progressed |
| | RTH | No change | | | | | and died |
| 18 | MVPP | Rx failed | | | | | |
| | CHOP | Rx failed | | | | | |
| | R/TH | Rx failed | | | | | Died |
| 19 | VAPEC-B (ps) | Rx failed | | | | | Progressed |
| | Palliative RTH | | | | | | and died |
| 20 | MACOP | Rx failed | | | | | |
| | VP16+ara-c | Rx failed | | | | | |
| | HD MTX | Rx failed | | | | | |
| | RTH | Rx failed | | | | | Died |
| 21 | MACOP | Rx failed | | | | | |
| | VAPEC-B (ps) | Rx failed | | | | | Progressed |
| | VAPEC B→ MACOP | | | | | | and died |
| 22 | VAPEC B | Inevaluable for response, died within 72 h | | | | | |

Key: RTH, radiotherapy; Rec, recurrence; WWP, well without progression; Rx, treatment; H/D, high dose. MACOP, adriamycin, cyclophosphamide, vincristine and prednisolone+mid-cycle methotrexate (ref); VAPEC-B, a 12 week sequential regimen comprising adriamycin, cyclophosphamide, vincristine and prednisolone+etoposide and bleomycin (ref); VAPEC-B ps, pilot study for the above regimen, bleomycin being given by i.v. infusion over 24 h; VP16, etoposide; ara-C, cytosine arabinoside.

Entries in bold indicate maximum response achieved for each patient.

One patient developed MLCLS 1½ years after receiving chemotherapy for documented stage IIB Hodgkin's disease.

*Definitions*

Clinical stage was defined on the basis of physical examination, full blood count, liver function tests, bone marrow aspirate and trephine biopsy, chest radiograph and computed tomographic (CT) scans of the chest and abdomen. 'Bulky' disease was defined as a mediastinal mass measuring $> 1/3$ of the internal thoracic diameter at D5/6.

Response was defined as follows:

Complete remission (CR): disappearance of all detectable signs of disease.

Good partial remission (GPR): no clinical evidence of disease but minimal residual abnormalities of uncertain significance persisting on radiological investigation.

Poor partial remission (PPR): a reduction in measurable disease of at least 50%.

Any lesser degree of response was deemed 'treatment failure'.

Duration of remission and overall survival were calculated according to the Kaplan–Meier method (Kaplan & Meier, 1973).

*Treatment*

Details of treatment are shown in Table II. The initial treatment reflects the protocols for high-grade lymphoma in use at the time (Whelan *et al.*, 1992; Dhaliwal *et al.*, 1993; Radford *et al.*, 1993). When this did not result in complete remission being achieved (because of lack of response or an incomplete response), alternative treatment (radiotherapy or chemotherapy) was given in the hope of eventually achieving CR. Thus, in Table II 'initial and subsequent treatment' refers to the treatment or consecutive treatments given in an attempt to achieve the maximum response. The four most recently treated patients have electively received radiotherapy (4,000 cGy) to the mediastinum on completion of chemotherapy, following reports in the literature which suggested improved survival with combined modality therapy (Jacobson *et al.*, 1988; Todeschini *et al.*, 1990; Al-Sharabati *et al.*, 1991).

### Results

*Response to therapy*

The response to each phase of treatment and the maximum response achieved are shown in detail in Table II. Complete remission was achieved with the initial chemotherapy in only 4/22 patients, GPR in seven and PPR in four, giving an overall response rate of 15/22 (68%). Seven patients showed no evidence of response, one being moribund on arrival at hospital; despite treatment being commenced, she died within 48 h.

Sixteen of the 17 patients in whom anything less than CR was achieved subsequently received additional, alternative therapy (one chemotherapy, six mediastinal radiotherapy, nine both treatment modalities, Table II). However, in only two did this result in a further documented radiological response, i.e. GPR becoming CR. In two of the seven patients in whom the initial treatment demonstrably failed, the use of mediastinal radiotherapy or etoposide + high-dose cytosine arabinoside (Whelan *et al.*, 1992) resulted in GPR and PPR being achieved eventually, but in both patients the response was short-lived and both subsequently died. Thus, if maximal rather than initial response is considered, the overall response rate becomes 17/22 (77%) (six CR, six GPR, five PPR). There were no treatment-related deaths.

*Duration of remission (Figure 1)*

With a median follow-up of 5½ years, 11/22 patients (5/6 CR, 3/6 GPR, 3/5 PPR) remain well without progression between 6 months and 14 years.

*Site of recurrence*

In all patients who developed recurrent disease, the predominant site was again the mediastinum.

*Survival (Figure 1)*

Twelve of the 22 patients remain alive, one having developed progressive disease despite additional radiotherapy and having subsequently received further, alternative chemotherapy. Ten of the 13 patients presenting with stage II/IIE disease are alive, compared with only two of the nine who had stage IV disease at presentation.

### Discussion

These results illustrate the experience of a single centre in the management of a rare subtype of high-grade B-cell lymphoma. The patients were not uniformly treated, reflecting developments in treatment approach. Although the number of patients is small, the results clearly demonstrate that a proportion of patients in whom this diagnosis is made are potentially curable with chemotherapy alone. However, in the majority, complete remission was not achieved with the original treatment; whether it would have been had more intensive initial therapy been used is debatable.

It has been suggested that therapy such as MACOP-B (Klimo & Connors, 1985) improves both remission rate and long-term outcome in patients with this diagnosis (Jacobson *et al.*, 1988; Todeschini *et al.*, 1990; Bertini *et al.*, 1991); it has also been reported that radiotherapy, given as consolidation of first remission, is advantageous (Jacobson *et al.*, 1988; Todeschini *et al.*, 1990; Al-Sharabati., 1991). While the latter contention cannot be proved in this series, half of the patients who remain well without progression did in fact receive radiotherapy at some point (Table II). In two patients in whom GPR was achieved with the original chemotherapy, radiotherapy did result in a further degree of response, at least by radiological criteria (gallium scans were not performed). On the other hand, in four patients, radiotherapy did not result in any further radiological response.

In patients with lymphoma, it is well established that a residual mediastinal shadow following chemotherapy may not necessarily represent active disease. This observation has most frequently been made in patients with Hodgkin's disease (Jochelson *et al.*, 1985; Chen *et al.*, 1987; Radford *et al.*, 1988) but is also true for patients with high-grade non-Hodgkin's lymphoma (Dhaliwal *et al.*, 1993). In this series, the presence of a residual mediastinal abnormality after treatment certainly did not necessarily imply treatment failure, provided at least a partial (>50%) response had been achieved. This is in contrast to the situation in patients in whom the initial therapy demonstrably failed, in whom the prognosis was extremely grave. In the latter group, tenuous responses to one or other chemotherapy regimen were sometimes seen, only to be rapidly overtaken by relentless progression of, almost invariably, intrathoracic rather than disseminated disease. The use of myeloablative therapy with autologous bone marrow transplantation might arguably have helped such patients, but on the two occasions in which it was used recurrent lymphoma supervened within months. Thus, although this is a tumour with a recognisable histological subtype and a typical clinical presentation, the situation for patients with MLCLS overall is no different from that of patients with other types of high-grade B-cell lymphoma. The difference lies in the distribution of disease at presentation, recurrence and progression when, almost invariably, the predominant site remains the mediastinum.

We are pleased to acknowledge the help of the medical and nursing staff of the Bodley Scott Unit and the staff of Haematology, Radiology and Radiotherapy at St Bartholomew's Hospital. We thank Claire Hole for typing the manuscript.

### References

ADDIS, B.J. & ISAACSON, P.G. (1986). Large cell lymphoma in the mediastinum: a B-cell tumour of probable thymic origin. *Histopathology*, **10**, 379–390.

AL-SHARABATI, M., CHITTAL, S., DUGA-NEEULAT, I., LAURENT, G., MAZEROLLES, C., AL-SAATI, T., BROUSSET, P. & DELSOL, G. (1991). Primary anterior mediastinal B-cell lymphoma. A clinicopathologic and immunohistochemical study of 16 cases. *Cancer*, **67**, 2579–2587.

BERTINI, M., ORSUCCI, L., VITOLO, U., LEVIS, A., TODESCHINI, G., MENEGHINI, V., NOVERO, D., TARELLA, C., GALLO, E., LUXI, G., PIZZUTI, M., NOVARINO, A., URGESI, A. & RESEGOTTI, L. (1991). Stage II large B-cell lymphoma with sclerosis treated with MACOP-B. *Ann. Oncol.*, **2**, 733–737.

CHEN, J.L., OSBORNE, B.M. & BUTLER, J.J. (1987). Residual fibrous masses in treated Hodgkin's disease. *Cancer*, **60**, 407–412.

DHALIWAL, H.S., ROHATINER, A.Z.S., GREGORY, W., RICHARDS, M.A., JOHNSON, P.W.M., WHELAN, J.S., GALLAGHER, C.J., MATTHEWS, J., GANESAN, T.S., BARNETT, M.J., WAXMAN, J.H., STANSFELD, A.G., WRIGLEY, P.F.M., SLEVIN, M.L., MALPAS, J.S. & LISTER, T.A. (1993). Combination chemotherapy for intermediate and high grade non-Hodgkin's lymphoma. *Br. J. Cancer*, **68**, 767–774.

ISAACSON, P.G., NORTON, A.J. & ADDIS, B.J. (1987). The human thymus contains a novel population of B lymphocytes. *Lancet*, **ii**, 1488–1491.

JACOBSON, J.O., AISENBERG, A.C., LAMARRE, L., WILLETT, C., LINGOOD, R., MIKETIC, L. & HARRIS, N. (1988). Mediastinal large cell lymphoma. An uncommon subset of adult lymphoma curable with combined modality therapy. *Cancer*, **62**, 1893–1898.

JOCHELSON, M., MAUCH, P., BALIKIAN, J., ROSENTHAL, D. & CANELLOS, G. (1985). The significance of the residual mediastinal mass in treated Hodgkin's disease. *J. Clin. Oncol.*, **3**, 637–640.

KAPLAN, E.L. & MEIER, P. (1973). Non parametric estimation from incomplete observations. *Am. Stat. Assoc. J.*, **53**, 457–481.

KLIMO, P. & CONNORS, J.M. (1985). CHOP-M-B chemotherapy for the treatment of diffuse large cell lymphoma. *Ann. Int. Med.*, **102**, 596–602.

LAMARRE, L., JACOBSON, J.O., AISENBERG, A.C. & HARRIS, N.L. (1989). Primary large cell lymphoma of the mediastinum. A histologic and immunophenotypic study of 29 cases. *Am. J. Surg. Pathol.*, **13**, 730–739.

LISTER, T.A. (1991). The management of follicular lymphoma. *Ann. Oncol*, **2**, 131–135.

MENESTRINA, F., CHILOSI, M., BONETTI, F., LESTANI, M., SCARPA, A., NOVELLI, P., DOGLIONI, C., TODESCHINI, G., AMROSETTI, A. & FIORE-DONATI, L. (1986). Mediastinal large-cell lymphoma of B-type, with sclerosis: histopathological and immunohistochemical study of eight cases. *Histopathology*, **10**, 589–600.

MILLER, J.B., VARIAKOJIS, D., BITRAN, J.D., SWEET, D., KINZIE, J., GOLOMB, H. & ULTMANN, J. (1981). Diffuse histiocytic lymphoma with sclerosis: a clinicopathologic entity frequently causing superior venacaval obstruction. *Cancer*, **47**, 748–756.

PERRON, T., FRIZZERA, G. & ROSAI, J. (1986). Mediastinal diffuse large-cell lymphoma with sclerosis. A clinicopathologic study of 60 cases. *Am. J. Surg. Pathol.*, **10**, 176–191.

RADFORD, J.A., COWAN, R.A. & FLANAGAN, M. (1988). The significance of residual mediastinal abnormality on the chest radiograph following treatment for Hodgkin's Disease. *J. Clin. Oncol.*, **6**, 940–946.

RADFORD, J.A., WHELAN, J.S., DEAKIN, D., HARRIS, M., STANSFELD, A.G., SWINDELL, R., WILKINSON, P.M.W., JAMES, R.D., LISTER, T.A. & CROWTHER, D. (1993). Weekly VAPEC-B chemotherapy for high grade non-Hodgkin's lymphoma: results of treatment in 184 patients. *Ann. Oncol.*, (in press).

SCARPA, A., BONETTI, F., MENESTRINA, F., MANEGAZZI, M., CHILOSI, M., LESTANI, M., BOVOLENTA, C., ZAMBONI, G. & FIORE-DONATI, L. (1987). Mediastinal large-cell lymphoma with sclerosis: genotypic analysis establishes its B nature. *Virchows Arch. A.*, **412**, 17–21.

SCARPA, A., BORGATO, L., CHILOSI, M., CAPELLI, P., MENESTRINA, F., BONETTI, F., ZAMBONI, G., PIZZOLO, G., HORHASHI, S. & FIORE-DONATE, L. (1991). Evidence of c-*myc* gene abnormalities in mediastinal large B-cell lymphoma of young adult age. *Blood*, **78**, 780–788.

TODESCHINI, G., AMBROSETTI, A., MENEGHINI, G., PIZZOLO, F., MENESTRINA, F., CHILOSI, M., BENEDETTI, F., VENERI, D., CETTO, G.L. & PERONA, G. (1990). Mediastinal large-B-cell lymphoma with sclerosis: a clinical study of 21 patients. *J. Clin. Oncol.*, **8**, 804–808.

TRUMP, D.L. & MANN, R.B. (1982). Diffuse large cell and undifferentiated lymphomas with prominent mediastinal involvement. A poor prognostic subset of patients with non-Hodgkin's lymphoma. *Cancer*, **50**, 277–282.

WHELAN, J.S., DAVIS, C.L., LEAHY, M., MACCALLUM, P., GUPTA, R.K., NORTON, A.J., AMESS, J.A.L., ROHATINER, A.Z.S. & LISTER, T.A. (1992). Intermediate dose Cytosine arabinoside in combination with Etoposide given with the intention of myeloablative consolidation therapy for refractory and recurrent haematological malignancy. *Haematol. Oncol.*, **10**, 87–94.

YOUSEM, S.A., WEISS, L.M. & WARNKE, R.A. (1985). Primary mediastinal non-Hodgkin's lymphomas: a morphologic and immunologic study of 19 cases. *Am. J. Clin. Pathol.*, **83**, 676–680.